# Contamination of aflatoxin B_1_, deoxynivalenol and zearalenone in feeds in China from 2021 to 2024

**DOI:** 10.1186/s40104-025-01213-w

**Published:** 2025-05-09

**Authors:** Meng Liu, Zhiyuan Xia, Yu Zhang, Rengui Yang, Weicai Luo, Lijia Guo, Ying Liu, Dessalegn Lamesgen, Hua Sun, Jiangfeng He, Lvhui Sun

**Affiliations:** 1 Inner Mongolia Academy of Agriculture and Animal Husbandry Science, Hohhot, Inner Mongolia 010031 China; 2 State Key Laboratory of Agricultural Microbiology, Hubei Hongshan Laboratory, Frontiers Science Center for Animal Breeding and Sustainable Production, College of Animal Science and Technology, Huazhong Agricultural University, Wuhan, Hubei 430070 China; 3 Tang Ren Shen Group Co., Ltd., Zhuzhou, Hunan 412007 China; 4 Haixing Group Co., Ltd., Zhangzhou, Fujian 363100 China; 5 Hebei Panshuo Biotechnology Co., Ltd., Baoding, Hebei 071599 China; 6 Tianjin Animal Disease Prevention and Control Center, Tianjin, 300402 China

**Keywords:** Aflatoxin B_1_, China, Contamination, Deoxynivalenol, Feeds, Zearalenone

## Abstract

**Background:**

This study was carried out to investigate the individual and combined contamination of aflatoxin B_1_ (AFB_1_), deoxynivalenol (DON), and zearalenone (ZEN) in feeds in China between 2021 and 2024. A total of 23,003 feed samples, including 17,489 feedstuff samples and 5,514 complete feed samples, were collected from different provinces of China for mycotoxin analysis.

**Results:**

The analyzed mycotoxins displayed considerably high contamination in the feed samples, with the individual contamination of AFB_1_, DON, and ZEN were 20.0%–100%, 33.3%–100%, and 85.0%–100%, respectively. The average concentrations of AFB_1_, DON, and ZEN were 1.2–728.7 μg/kg, 106–8,634.8 μg/kg, and 18.1–3,341.6 μg/kg, respectively. Notably, the rates over China’s safety standards for AFB_1_, DON, and ZEN in raw ingredients were 9.7%, 2.7%, and 15.7%, respectively. Meanwhile, 3.5%, 1.1%, and 8.7% of analyzed complete feeds exceeded China’s safety standards for AFB_1_, DON, and ZEN, respectively. Moreover, the co-contamination rates of AFB_1_, DON, and ZEN in more than 70% of raw ingredients and 87.5% of complete feed products were 60.0%–100% and 61.5%–100%, respectively.

**Conclusion:**

This study reveals that the feeds in China have commonly been contaminated with AFB_1_, DON, and ZEN alone and their combination during the past four years. These findings highlight the significance of monitoring mycotoxin contaminant levels in domestic animal feed and the importance of carrying out feed administration and remediation strategies for mycotoxin control.

## Background

Mycotoxins are naturally occurring toxic secondary metabolites of fungi, primarily produced by five genera, including *Aspergillus*, *Alternaria*, *Claviceps*, *Fusarium*, and *Penicillium* [1–4]. As of the latest research, over 500 mycotoxins have been identified, posing a significant threat to human and animal health [5]. Among the most prevalent mycotoxins found in agricultural commodities are aflatoxin B_1_ (AFB_1_), deoxynivalenol (DON), and zearalenone (ZEN) [6–8]. AFB_1_, produced mainly by *Aspergillus*, is the most toxic mycotoxin, exhibiting hepatotoxic, carcinogenic, mutagenic, and teratogenic properties in various animal species [9–14]. Both DON and ZEN are primarily produced by *Fusarium* [15–17]. DON, a type B trichothecene, can induce anorexia, vomiting, and intestinal and immune system disorders in multiple species by inhibiting DNA, RNA, and protein synthesis [18–22]. ZEN is an estrogenic mycotoxin as it competes with 17β-estradiol for estrogen receptor binding, and can induce reproductive and fertility disorders [23–27].

Since mycotoxins in feed can negatively affect animal health, but can also threaten human health when converted into animal products, many countries have established safety standards for these toxins in feed and feed ingredients. For example, the European Commission set the maximum levels of AFB_1_, DON, and ZEN at 5–20, 900, and 250 μg/kg, respectively, in feed ingredients and complete feed [28, 29]. In 2017, General Administration of Quality Supervision, Inspection and Quarantine of the People’s Republic of China released the latest version of safety standards (GB 13078–2017) for AFB_1_, DON, and ZEN; which are 10–20, 1,000–5,000 and 100–250 μg/kg, respectively, for feedstuffs and complete feeds [7, 30].

However, current global warming and climate change are making corn, wheat and other crops more susceptible to fungal colonization, and mycotoxin contamination [31–33]. Thus, it will be important to monitor mycotoxin levels in livestock feed ingredients well into the future to maintain animal health and ensure the safety of human food products. There are several climatic regions across China, especially the warm or humid conditions of the Yangtze, Yellow River basins and northeast region and their numerous rainfall events, are favorable for mold growth and mycotoxin production in crops, which is increasing crop susceptibility to fungal infection [34–36]. Therefore, monitoring mycotoxin concentrations in the feedstuffs and complete feeds from these and other regions across China is essential to prevent farm animal exposure to mycotoxins and to ensure feed and food safety. Thus, the current study was conducted to investigate and renew the individual and combined contamination of AFB_1_, DON, and ZEN in more than 23,000 feedstuffs and complete feeds collected from different regions of China during the past 4 years.

## Methods

### Sample collection and preparation

A total of 23,003 feed samples were collected from 2021 to 2024 from either companies or livestock farms in different regions of China. There were 17,489 feedstuff samples including 7,018 corn, 1,558 dried distillers grains with solubles (DDGS), 1,608 corn gluten meal, 968 corn germ meal, 42 corn bran, 1,734 wheat, 845 wheat flour, 340 wheat bran, 169 wheat middlings, 44 wheat germ, 2,558 peanut meal, 58 cottonseed meal, 49 soybean meal, 82 brown rice, 24 rice bran, 20 rice bran meal, 36 sorghum, 182 distiller's grains, 80 corn sugar residue, 33 sugar residue, 27 nucleotide residue, 14 cottonseed protein, along with 5,514 complete feed samples including 658 pig feed, 4,814 poultry feed and 42 ruminant feed. These feed samples were primarily collected from the provinces of Anhui, Beijing, Chongqing, Fujian, Guangdong, Guangxi, Gansu, Henan, Hebei, Hunan, Hubei, Heilongjiang, Inner Mongolia, Jiangsu, Jiangxi, Jilin, Liaoning, Ningxia, Shandong, Sichuan, Shanxi, and Zhejiang by the feed companies. Since few feed samples with insufficient quantity, 11,826, 14,812, and 8,794 samples were analyzed for AFB_1_, DON, and ZEN, respectively. The feed samples were stored in sealing bags at −20 °C before analysis.

### Extraction and quantified of AFB_1_, DON and ZEN

The extraction and quantified of AFB_1_, DON, and ZEN from the feed samples collected between 2021 and 2024 were performed as previously described [7, 35–39]. Briefly, 5 g of the mashed feed samples were mixed with a 25-mL solution of ethanol:water (50:50, v/v), blended using a commercial blender at high speed for 3 min, centrifuged at 4,200 r/min for 10 min, filtrated and collected the filtrate. The quantification of AFB_1_, DON, and ZEN were performed with commercially available test kits purchased from TRUTHERS Biotechnology Co., Ltd. (Tianjin, China) according to their operating instructions. Specially, the quantum dot fluorescence immunochromatographic assay were used to detection. The mycotoxins in the sample bind to the specific antibodies labeled with quantum dots, inhibiting the binding between the antibodies and the mycotoxin-BSA conjugate on the detection line (T line), resulting in changes in the fluorescence value of the T line. The content of mycotoxins is calculated by the high and low fluorescence value signals of the T line. The limit of detection (LOD) for AFB_1_, DON and ZEN set at 1.0 μg/kg, 100 μg/kg and 10 μg/kg, respectively.

### Statistical analysis

All the data were analyzed by Microsoft Excel 2019 (Microsoft Corporation, Redmond, USA) and expressed as means, median, maximum, or percentages.

## Results

### Contamination of AFB_1_ in feeds

A total of 11,826 feed samples, including 8,442 feedstuffs and 3,384 complete feeds, were collected between 2021 to 2024 for analysis of AFB_1_ (Table 1). AFB_1_ was detected in 20.0%–100% of feedstuff and complete feeds, with the average levels ranging from 1.2 to 728.7 μg/kg. The highest median concentration of AFB_1_ was 770 μg/kg in wheat bran from the 2024 harvest, followed by 390 μg/kg in complete pig feed and 185.2 μg/kg in corn sugar residue from the 2024 harvest. The maximum levels of AFB_1_ were 4,490 μg/kg in DDGS harvested in 2024, followed by 2,380 μg/kg in wheat and 1,720 μg/kg in corn harvested in 2024. Among all the analyzed feedstuff samples, 819 raw feed ingredient samples (accounting for 9.7%) were contaminated with AFB1 at concentrations exceeding China’s safety standard. Notably, 74 samples of complete pig feed and 43 samples of complete poultry feed, which account for 2.2% and 1.3% of all the analyzed complete feed samples, were contaminated with AFB_1_ at levels exceeding the Chinese safety standard concentrations.

**Table 1:** AFB_1_ concentrations in feeds^a^

Item	Year	Number of samples	Positive samples, μg/kg				Over standard rate, %
			%	Mean	Median	Maximum	
Corn	2021	636	66.7	7.5	2.4	95.6	1.6
	2022	1,007	70.9	10.9	3.7	214.0	2.3
	2023	335	83.9	10.6	3.0	180.8	3.3
	2024	757	61.3	80.1	3.7	1,720	7.9
Dried distillers grains with solubles	2021	175	82.9	4.0	2.8	24.7	0
	2022	149	77.2	6.0	2.2	38.1	0
	2023	71	80.3	8.1	2.3	93.1	2.8
	2024	460	90.9	136.4	15.8	4,490	3.5
Corn gluten meal	2021	408	92.6	17.7	7.2	806.0	6.1
	2022	274	90.5	11.0	5.8	122.3	1.8
	2023	163	94.5	19.7	7.5	189.4	11.0
	2024	146	100	32.8	23.9	167.8	16.4
Corn germ meal	2021	278	98.2	9.2	7.4	37.7	0
	2022	63	92.1	7.1	5.3	25.5	0
	2023	45	95.6	24.4	17.4	119.1	13.3
	2024	385	87.8	18.5	5.8	650	0.3
Corn bran	2021	12	100	7.7	5.0	26.8	0
	2022	9	100	14.8	4.1	91.1	11.1
	2023	-	-	-	-	-	-
	2024	1	100	24.8	24.8	24.8	0
Wheat	2021	160	72.5	3.1	2.1	37.5	0.6
	2022	5	20.0	1.2	1.2	1.2	0
	2023	43	76.7	2.8	1.8	17.9	0
	2024	13	84.6	742.1	6.9	2,380	30.8
Wheat flour	2021	41	61.0	2.7	2.0	9.0	0
	2022	4	0	ND	ND	ND	0
	2023	21	42.9	1.2	1.2	1.6	0
	2024	2	100	3.5	3.5	5.6	0
Wheat bran	2021	26	69.2	3.8	2.7	9.5	0
	2022	10	90.0	1.8	2.1	2.6	0
	2023	2	100	4.7	4.7	4.7	0
	2024	7	85.7	850.0	770.0	1,610	85.7
Peanut meal	2021	1,116	99.1	31.3	26.0	195	14.0
	2022	696	95.8	58.9	35.2	617.3	32.9
	2023	433	98.4	46.2	28.0	417.8	30.5
	2024	220	98.6	46.7	25.6	254.1	32.3
Cottonseed meal	2021	22	90.9	5.1	3.5	13.5	0
	2022	13	92.3	6.7	2.9	28.4	0
	2023	14	100	5.6	4.5	15.9	0
	2024	3	100	14.8	17.8	20.2	0
Soybean meal	2021	21	90.5	2.7	2.0	15.9	0
	2022	5	60.0	1.9	2.2	1.4	0
	2023	-	-	-	-	-	-
	2024	2	100	9.9	9.9	10.7	0
Brown rice	2021	30	66.7	2.2	2.2	5.2	0
	2022	12	66.7	2.1	2.1	3.4	0
	2023	3	66.7	1.3	1.3	1.6	0
	2024	-	-	-	-	-	-
Distiller's grains	2021	10	90.0	4.0	2.9	14.7	0
	2022	28	100	15.0	4.7	90.9	17.9
	2023	28	89.3	9.3	4.2	43.1	32.1
	2024	3	100	43.5	44.4	45.5	0
Corn sugar residue	2021	11	63.6	2.6	2.8	4.5	0
	2022	26	80.8	2.1	1.8	4.3	0
	2023	4	100	4.8	3.6	10.7	0
	2024	1	100	185.2	185.2	185.2	100
Sugar residue	2021	-	-	-	-	-	-
	2022	10	90.0	2.2	2.0	3.9	0
	2023	10	100	10.8	11.2	17.3	0
	2024	2	100	7.7	7.7	13.6	0
Nucleotide residue	2021	10	100	6.0	4.6	16.9	0
	2022	1	100	3.0	3.0	3.0	0
	2023	-	-	-	-	-	-
	2024	-	-	-	-	-	-
Complete pig feed	2021	42	95.2	3.8	5	5.8	0
	2022	73	28.8	3.5	2.8	15.3	1.3
	2023	33	81.8	7.3	1.7	133.8	3.0
	2024	83	100	376.4	390	810	85.5
Complete poultry feed	2021	948	86.9	3.6	2.8	63.7	0.7
	2022	867	83.9	4.9	3.5	21.4	1.4
	2023	829	87.8	6.0	2.9	774.8	1.9
	2024	488	95.5	5.5	4.2	78.9	1.6
Complete ruminant feed	2021	3	100	1.7	1.8	2.3	0
	2022	4	75	1.9	1.9	2.6	0
	2023	12	66.7	1.6	1.6	2.1	0
	2024	2	100	1.8	1.8	2.3	0

^a^Positive samples are defined as those with AFB_1_ ≥ 1.0 μg/kg (LOD)

“-” represents that no samples of this feed type were collected. *ND* Not detected

### Contamination of DON in feeds

In total, 14,812 feed samples, including 11,527 feedstuff and 3,285 complete feeds, were collected between 2021 to 2024 for analysis of DON (Table 2). DON was detected in 33.3%–100% of feedstuff and complete feeds, with the average levels ranging from 106 to 8,634.8 μg/kg. The highest median concentration of DON was 9,762 μg/kg in corn bran from the 2021 harvest, followed by 7,177 μg/kg and 4,840 μg/kg in corn germ meal from the 2023 and 2022 harvests. The maximum levels of DON were 84,000 μg/kg in wheat flour harvested in 2023, followed by 53,860 μg/kg in corn harvested in 2023 and 34,800 μg/kg in corn germ meal harvested in 2022. Among all the analyzed feedstuff samples, 316 raw feed ingredient samples (accounting for 2.7%) were contaminated with DON at concentrations over 5,000 μg/kg. Notably, the content of DON in corn germ meal and DDGS harvested between 2021 to 2024 exceeded the standard by 9.1%–69.5% and 2.6%–21.2%. Additionally, 24 samples of complete pig feed, 7 samples of complete poultry feed, and 4 samples of complete ruminant feed were contaminated with DON at levels exceeding the Chinese safety standard concentrations, which accounted for 4.6%, 0.3% and 13.3% of complete feeds samples of the same category analyzed, respectively.

**Table 2:** DON concentrations in feeds^a^

Item	Year	Number of samples	Positive samples, μg/kg				Over standard rate, %
			%	Mean	Median	Maximum	
Corn	2021	1,302	97.7	1,071.2	800.0	16,359	0
	2022	2,171	96.5	1,316.5	1,000	9,830	0.8
	2023	659	91.2	1,062.6	659.8	53,860	0.6
	2024	1,252	89.4	1,009.2	802.5	4,698	0
Dried distillers grains with solubles	2021	455	100	2,627.8	2,600	6,413.4	2.6
	2022	326	100	3,839.1	3,651.7	15,670	21.2
	2023	120	95.0	2,905.0	2,651.5	9,366	10.0
	2024	482	94.0	2,065.3	1,747.0	13,586.2	2.7
Corn gluten meal	2021	206	97.1	1,112.4	710.5	4,932.8	0
	2022	229	96.9	1,848.4	1,420.0	9,750	3.9
	2023	119	94.1	1,084.5	655.5	8,080	1.7
	2024	128	93.8	944.6	915.0	3,264	0
Corn germ meal	2021	124	99.2	3,213.3	2,580.0	28,500	12.9
	2022	65	100	8,634.8	4,840	34,800	47.7
	2023	82	100	7,343.8	7,177	19,831	69.5
	2024	396	95.2	2,083.5	1,293.0	16,266	9.1
Corn bran	2021	5	100	8,344.9	9,762	10,720	100
	2022	8	100	4,005.7	3,491.8	6,996.2	25.0
	2023	-	-	-	-	-	-
	2024	14	100	2,597.9	2,689.8	3,540.5	0
Wheat	2021	1,354	97.0	1,419.0	1,180.0	7,200	0.6
	2022	109	96.3	1,257.3	880.0	4,880	0
	2023	134	93.3	690.4	510.0	2,660	0
	2024	54	87.0	792.5	431.0	3,670.4	0
Wheat Flour	2021	515	92.0	590.8	390.0	8,000	0.2
	2022	217	90.3	576.9	388.5	4,090	0
	2023	65	93.8	2,087.1	551.0	84,000	1.5
	2024	27	92.6	445.0	390.0	1,490.8	0
Wheat bran	2021	129	100	953.5	690	4,109.9	0
	2022	91	98.9	846.4	557.5	3,720	0
	2023	35	94.3	1,788.9	1,080.0	23,900	2.9
	2024	23	100	1,015.8	810	2,720	0
Wheat middlings	2021	88	100	1,244.8	1,000	3,760	0
	2022	58	100	1,683.9	1,100	5,380	6.9
	2023	8	100	1,258.5	795	3,460	0
	2024	11	100	1,617.8	560	4,182	0
Wheat germ	2021	23	95.6	611.5	355.0	5,000	0
	2022	14	92.9	341.4	322.0	587	0
	2023	-	-	-	-	-	-
	2024	1	100	653	653	653	0
Brown rice	2021	46	100	762.1	303.5	9,620	2.2
	2022	10	100	713.6	362.5	2,410	0
	2023	7	85.7	513.2	400.0	975	0
	2024	1	100	570	570	570	0
Peanut meal	2021	42	40.5	1,604.4	1,360.0	5,100	2.4
	2022	11	81.8	477.0	110.0	3,436	0
	2023	19	100	522.1	187.3	3,140	0
	2024	24	37.5	156.8	147.7	220	0
Distiller's grains	2021	34	94.1	3,007.5	2,637.1	7,343.4	5.9
	2022	36	100	2,943.5	3,035	5,560	5.6
	2023	30	93.3	2,978.9	2,532.6	8,560	6.7
	2024	9	100	4,410.8	3,833	11,270	22.2
Rice bran	2021	15	73.3	602.5	278.0	1,740	0
	2022	1	100	106	106	106	0
	2023	1	100	250	250	250	0
	2024	1	100	678	678	678	0
Rice bran meal	2021	12	91.7	341.9	305.0	7,170	0
	2022	1	100	241	241	241	0
	2023	-	-	-	-	-	-
	2024	1	0	ND	ND	ND	0
Soybean meal	2021	11	63.6	623.7	856.7	1,081.8	0
	2022	8	75.0	731.4	878.3	1,145.3	0
	2023	2	0	ND	ND	ND	0
	2024	-	-	-	-	-	-
Sorghum	2021	15	80.0	997.0	204.5	3,700	0
	2022	9	33.3	578.3	624.0	810	0
	2023	2	0	ND	ND	ND	0
	2024	2	50.0	3,078.0	3,078.0	3,078	0
Corn sugar residue	2021	8	87.5	1,651.4	1,460.0	4,008	0
	2022	55	100	2,925.3	2,677	7,880	9.1
	2023	4	100	1,245	821.5	2,897	0
	2024	-	-	-	-	-	-
Complete pig feed	2021	268	97.0	386.7	306.8	2,258.6	5.2
	2022	136	50.0	457.0	212.4	2,031.2	6.6
	2023	71	97.2	364	334.9	800	0
	2024	48	97.9	394.2	350	1,336.5	2.1
Complete poultry feed	2021	460	95.9	737.4	550	5,377.8	0.4
	2022	472	98.7	709	750	1,550	0
	2023	1,037	97.9	737.7	603	4,800	0.4
	2024	663	96.3	824.3	719	3,229	0.15
Complete ruminant feed	2021	3	100	885.9	968.4	970.7	0
	2022	2	100	396.4	396.4	614.2	0
	2023	17	76.5	318.2	276.0	716.9	23.5
	2024	8	62.5	488.8	478.6	832.2	0

^a^Positive samples are defined as those with DON ≥ 100 μg/kg (LOD)

"-" represents that no samples of this feed type were collected. *ND* Not detected

### Contamination of ZEN in feeds

A total of 8,794 feed samples, including 6,153 feedstuffs and 2,641 complete feeds, were collected between 2021 and 2024 for analysis of ZEN (Table 3). ZEN was detected in 85.0%–100% of feedstuff and complete feeds, with the average levels ranging from 18.1 to 3,341.6 μg/kg. The highest median concentration of ZEN was 2,566.4 μg/kg in corn gluten meal from the 2024 harvest, followed by 1,824.7 μg/kg in corn bran from the 2021 harvest and 1,424.4 μg/kg in distiller’s grains from the 2024 harvest. The maximum levels of ZEN were 30,154 μg/kg in corn gluten meal harvested in 2022, followed by 10,958 μg/kg in corn harvested in 2022 and 10,345.1 μg/kg in corn gluten meal harvested in 2024. A total of 965 raw feed ingredient samples, which account for 15.7% of all the analyzed feedstuff samples, were contaminated with ZEN at concentrations over the Chinese safety standard concentrations. Additionally, 149 samples of complete pig feed and 82 samples of complete poultry feed, which account for 5.6% and 3.1% of all the analyzed complete feed samples, were contaminated with ZEN at levels exceeding the Chinese safety standard concentrations.

**Table 3:** ZEN concentrations in feeds^a^

Item	Year	Number of samples	Positive samples, μg/kg				Over standard rate, %
			%	Mean	Median	Maximum	
Corn	2021	779	95.4	135.0	59.4	4,663.9	3.7
	2022	1,190	97.9	319.2	123.0	10,958	16.5
	2023	475	88.4	242.7	59.5	5,482.8	9.5
	2024	918	81.1	176.0	90	2,983.4	4.6
Dried distillers grains with solubles	2021	228	100	324.6	268.7	1,246.8	0
	2022	234	100	890.1	448.7	4,871	14.9
	2023	104	100	621.6	219.9	4,272.3	18.3
	2024	457	92.8	611.4	538	4,101.6	4.2
Corn gluten meal	2021	169	99.4	1,241.7	561.1	10,247	52.1
	2022	205	100	2,947.7	673.8	30,154	57.1
	2023	103	100	1,517.0	614.0	7,293.2	53.4
	2024	114	100	3,341.6	2,566.4	10,345.1	75.4
Corn germ meal	2021	60	100	556.6	469.4	3,247	40.0
	2022	32	100	1,773.9	885.5	9,494.4	71.9
	2023	46	100	812.8	926.8	2,167.5	67.4
	2024	378	96.6	488.1	315	4,144	28.6
Corn bran	2021	5	100	1,737.2	1,824.7	2,203.3	80.0
	2022	5	100	974.6	454.2	2,041.6	40.0
	2023	1	100	572.1	572.1	572.1	0
	2024	12	100	186.9	119.4	716.6	0
Wheat	2021	138	100	74.5	48.9	548.2	0
	2022	20	85.0	470.6	493.0	1,902	5.0
	2023	79	96.2	53.4	24.8	922.4	0
	2024	21	90.5	43.2	43.49	81.0	0
Wheat Flour	2021	13	100	125.9	74.8	317.3	0
	2022	5	100	20.6	18.7	38.9	0
	2023	26	100	24.1	19.1	48.6	0
	2024	4	100	21.2	23.9	26.6	0
Wheat bran	2021	25	96.0	94.6	50	852.3	0
	2022	31	90.3	1,016.6	626	6,124	29.0
	2023	23	100	611.3	430	1,846.0	34.8
	2024	2	100	23.8	13.8	16.2	0
Peanut meal	2021	10	100	115.1	44.7	311.6	0
	2022	8	100	27.4	22.4	90.0	0
	2023	13	100	35.3	28.8	72.9	0
	2024	3	100	18.1	17.9	25.1	0
Soybean meal	2021	14	100	84.5	67.3	222.1	0
	2022	8	100	77.1	48.9	246.2	0
	2023	4	100	132.1	159.5	188.7	0
	2024	-	-	-	-	-	-
Cottonseed meal	2021	3	100	98.5	76.7	159.6	0
	2022	10	100	39.0	27.1	83.4	0
	2023	1	0	ND	ND	ND	0
	2024	-	-	-	-	-	-
Brown rice	2021	27	100	76.2	6.7	468	0
	2022	8	100	140.0	83.2	538.9	0
	2023	9	100	273.7	88.3	1,668.8	11.1
	2024	1	100	22.6	22.6	22.6	0
Distiller's grains	2021	10	100	872.2	119.0	4,663.9	20.0
	2022	40	100	1,014.6	408.9	10,203.1	12.5
	2023	14	100	392.1	301.5	1,161.1	7.1
	2024	6	100	2,800.9	1,424.4	9,962.3	66.7
Corn sugar residue	2021	-	-	-	-	-	-
	2022	31	100	404.1	394.5	1,367.9	25.8
	2023	3	100	185.1	164.2	230.8	0
	2024	-	-	-	-	-	-
Sugar residue	2021	-	-	-	-	-	-
	2022	11	100	653.7	505.5	1,825.8	18.2
	2023	4	100	849.4	661.3	1,834.4	25.0
	2024	-	-	-	-	-	-
Complete pig feed	2021	84	92.9	72.7	50	3,307	13.1
	2022	143	100	542.2	554	1,175.0	85.3
	2023	76	100	110.2	52.7	849.3	17.1
	2024	81	100	64.7	44.5	229.7	3.7
Complete poultry feed	2021	373	99.2	143.9	102.1	975.2	2.1
	2022	581	100	315.4	363.6	2,497.7	8.8
	2023	783	99.9	146.0	107.0	774.8	0.25
	2024	483	99.8	232.5	195.1	1,003.2	4.3
Complete ruminant feed	2021	3	100	54.9	64.2	71.3	0
	2022	3	100	107.1	56.1	224.7	0
	2023	21	100	48.0	37.4	145.1	0
	2024	10	100	80.2	61.7	170.1	0

^a^Positive samples are defined as those with ZEN ≥ 10 μg/kg (LOD)

"-" represents that no samples of this feed type were collected. *ND* Not detected

### Co-contamination of AFB_1_, DON and ZEN in feeds

The co-contamination of AFB_1_, DON, and ZEN in feed samples between 2021–2024 was presented in Table 4. More than 70.0% of analyzed feed ingredient samples had a combined contamination rate of 60.0%–100% for AFB_1_ + DON, AFB_1_ + ZEN, DON + ZEN, and AFB_1_ + DON + ZEN. Notably, except for the complete pig feed collected from 2022, the co-contamination ranges of AFB_1_ + DON, AFB_1_ + ZEN, DON + ZEN, along with AFB_1_ + DON + ZEN in most complete feeds were 61.5%–100%, 76.7%–100%, 66.7%–100%, and 58.3%–100%, respectively.

**Table 4:** Percentage of AFB1, DON and ZEN co-occurrence in feeds^a^

Item	Year	AFB_1_ + DON, %	AFB_1_ + ZEN, %	DON + ZEN, %	AFB_1_ + DON + ZEN, %
Corn	2021	56.4	54.3	94.0	50.7
	2022	68.8	74.7	90.1	66.8
	2023	72.6	79.8	79.1	72.3
	2024	42.6	44.9	76.1	35.9
Wheat	2021	69.9	74.3	99.0	74.6
	2022	20.0	20.0	55.6	0
	2023	72.7	73.5	88.1	70.4
	2024	66.7	57.1	89.7	57.1
Dried distillers grains with solubles	2021	79.8	82.7	100	80.6
	2022	79.3	75.3	100	75.3
	2023	74.2	77.6	91.6	72.7
	2024	89.8	89.5	93.3	88.8
Corn gluten meal	2021	82.9	86.6	92.7	82.1
	2022	90.9	77.1	95.1	82.1
	2023	90.7	89.4	90	85.3
	2024	98.9	100	88.7	97.6
Corn germ meal	2021	93.2	92.1	100	88.0
	2022	91.4	89.5	94.7	88.2
	2023	93.1	90.9	100	90.9
	2024	85.0	86.9	94.4	84.6
Wheat bran	2021	68.2	61.5	91.7	61.5
	2022	100	83.3	61.5	83.3
	2023	100	100	70.0	100
	2024	-	-	100	-
Wheat flour	2021	48.4	50.0	91.7	0
	2022	-	-	100	-
	2023	40.0	50.0	95.5	37.5
	2024	100	-	75.0	-
Peanut meal	2021	31.3	100	50.0	83.3
	2022	80.0	100	-	-
	2023	100	100	100	100
	2024	31.8	100	66.7	50.0
Brown rice	2021	66.7	57.1	100	57.1
	2022	60.0	75.0	100	75.0
	2023	100	50.0	100	100
	2024	-	-	100	-
Distiller's grains	2021	88.9	100	100	100
	2022	88.9	87.5	100	87.5
	2023	85.7	100	90.0	100
	2024	100	100	100	100
Complete pig feed	2021	92.9	90.5	92.0	90.5
	2022	24.6	22.7	46.5	15.2
	2023	75.9	76.7	98.3	74.1
	2024	61.5	85.3	98.0	58.3
Complete poultry feed	2021	77.6	88.7	84.2	75.5
	2022	89.8	83.8	99.0	82.1
	2023	86.2	84.9	96.5	83.5
	2024	93.1	93.9	96.5	89.6
Complete ruminant feed	2021	100	100	100	100
	2022	100	100	100	100
	2023	62.5	80.0	73.3	71.4
	2024	100	100	66.7	100

^a^AFB_1_, aflatoxin B_1_; DON, deoxynivalenol; ZEN, zearalenone; AFB_1_ + DON, feeds co-contaminated with AFB_1_ and DON; AFB_1_ + ZEN, feeds co-contaminated with AFB_1_ and ZEN; DON + ZEN, feeds co-contaminated with DON and ZEN; AFB_1_ + DON + ZEN, feeds co-contaminated with AFB_1_, DON and ZEN

"-" represents no sample was analyzed for two or more toxins simultaneously

## Discussion

Mycotoxins have been reported to reduce animal performance, threaten human and animal health, as well as bring huge economic loss to the feed and food industry [4]. Thus, it will be important to monitor mycotoxin levels in livestock feed ingredients well into the future to maintain animal health and ensure the safety of human food products. Considering the widespread contamination, the present study was carried out to investigate the individual and combined contamination of the most prevalent and toxic mycotoxins, AFB_1_, DON, and ZEN in feedstuffs and complete feeds harvested between 2021 and 2024. In general, the analyzed mycotoxins displayed a considerably high occurrence in the analyzed feed samples harvested between 2021 and 2024, ranging from 20.0% to 100%, 33.3% to 100%, and 85.0% to 100% for AFB_1_, DON, and ZEN, respectively. Additionally, the LOD of the AFB_1_, DON, and ZEN are relatively high, which might underestimation of the actual exposure risk of these mycotoxins. These results indicated that the mycotoxin contamination of raw materials and complete feeds in China is quite serious.

The average concentration of AFB_1_ (1.2–728.7 μg/kg) determined in the present study was higher than previously reported concentrations (1.2–27.4 μg/kg) from samples harvested between 2016–2020 [7, 37]. Notably, 9.7% of all the analyzed raw feedstuff samples, especially corn, corn gluten meal, corn germ meal, and peanut meal, with AFB_1_ exceeded the Chinese safety standard concentration, which is much higher than the previously reported 0.9% of analyzed feedstuff samples with AFB_1_ exceeded the Chinese safety standard concentration [7, 30, 37]. Furthermore, there were 31.6% and 1.4% of the analyzed complete pig feed and poultry feed respectively contained AFB_1_, especially in complete pig feeds harvested in 2024, whereas the excess rate was as high as 85.5%. These results are much higher than the previous reports that 1.5% and 1.2% of all the analyzed final products for pig and poultry contained AFB_1_ over the limitation of Chinese safety standards [7]. The most toxic AFB_1_ is a frequent contaminant of various commodities, primarily cereals, nuts, and spices, and represents a highly toxic mycotoxin [9, 40, 41], therefore, it is necessary to persist in supervising the levels of AFB_1_ in the raw feed ingredients and its final products in the future. Additionally, since the safety limitation of AFB_1_ in European Commission (5–20 μg/kg) is lower than China (10–20 μg/kg), which might restrict the export of these Chinese feedstuffs [28–30].

The average levels of DON in feeds ranged from 106 to 8,634.8 μg/kg in this study, which is relatively higher than the previously reported range of 364.5–4,381.5 μg/kg in the feeds collected in China between 2013 and 2020 [7, 34, 37]. The maximum value of DON in feedstuffs in the current study is 84,000 μg/kg, which is at least 39 times higher than the values in all the reported areas [42]. There are 2.7% of the analyzed raw feed ingredients with DON exceeded China’s safety standard, especially as much as corn (9.1%–69.5%) and DDGS (2.6%–21.2%), which are much higher than the previously reported in 2013–2020 [7, 37]. Although only 4.6% of the complete pig feed samples exceeded the Chinese safety standard concentration, 0.3% of the complete poultry feed samples and 13.3% of the complete ruminant feed samples contaminated DON over the limitation of Chinese safety standard. These divergences could be attributed to the fact that the analyzed feed samples were randomly collected from different regions, and weather varies in these areas during the harvest period [7, 34, 35, 37, 43]. Taken together, these findings remind us that we need to be cognizant of the potential for contamination of feed ingredients, especially corn, DDGS, corn gluten meal, and corn germ meal, which were relatively severely contaminated by DON. In addition, since the safety limitation of DON in European Commission (900 μg/kg) is lower than China (1,000–5,000 μg/kg), which might restrict the export of these Chinese feedstuffs [28–30].

The occurrence of ZEN (85.0%–100%) in the analyzed feeds in this study was lower than previously study (96.9%–100%) from harvests in 2018–2020, but it was higher than feed samples (50.0%–100%) collected from 2013 to 2017 [7, 34, 35]. Meanwhile, in the current study, the average concentration of ZEN (18.1–3341.6 μg/kg) was relatively higher than previously reported results (48.1–326.8 μg/kg and 0–729.2 μg/kg) from harvests between 2013 and 2020 [7, 34, 35]. These differences might be due to the number of feed samples, different sampling areas, and different weather conditions during harvest. Notably, 15.7% of analyzed feedstuff samples, mainly including corn and its by-products, as well as 8.7% of analyzed complete feed samples (complete pig feed and complete poultry feed), were contaminated with ZEN at concentrations that exceeded the Chinese safety standards. These findings were much higher than previously reported, where 0.5% and 1.9% of all the analyzed feedstuffs and complete feed samples, respectively, were shown to be contaminated with ZEN exceeding the regulatory limits [7].

The co-occurrence and interaction between two or more mycotoxins were regularly found, and results suggested that co-contaminates can exhibit stronger toxic effects on animals when compared to each mycotoxin [44–47]. It is worth noting that the co-contamination of AFB_1_, DON, and ZEN, can exert additive and synergistic toxic effects on animal health and production [48]. In the present study, the co-contamination of mycotoxins in all analyzed feeds harvested in 2021–2024 was quietly universal, with more than 20% of feedstuff samples and 15.2% of complete feeds containing 2 or more mycotoxins. Notably, DDGS, corn gluten meal, corm germ meal, distiller’s grains, complete pig feed, and complete poultry feed were more than 70% co-contaminated with AFB_1_, DON, and ZEN. These results were similar to previous studies that mycotoxin contamination is a widespread issue in the livestock and poultry industry [48–52]. The present feed safety regulations do not consider the potential toxicity of co-contamination of mycotoxins and their combined toxicity on animal health and production may be underestimated. Therefore, the combined toxicity of these mycotoxins warrants further study, which might further reduce thresholds of the regulatory limits for mycotoxins. Although how to set the regulatory limits for mycotoxins under their co-contamination is very complicate, it still might be very important to consider it when new regulatory frameworks for mycotoxins are plan in the future.

## Conclusion

In summary, the mycotoxin survey of 23,003 samples collected from different areas of China from 2021 to 2024 indicated that mycotoxins are ubiquitously present in feeds. Generally, 9.7%, 2.7%, and 15.7% of analyzed raw feed ingredients collected between 2021 and 2024 were contaminated with AFB_1_, DON, and ZEN by exceeding the Chinese safety standards, respectively. Meanwhile, 3.4%, 1.1% and 8.7% of analyzed complete feeds exceeded China’s safety standards for AFB_1_, DON and ZEN, respectively. Moreover, it is worth noting that co-occurrence ≥ 2 mycotoxins were quite common in all analyzed feed samples. The co-contamination rates of the three mycotoxins (AFB_1_, DON and ZEN) in more than 70% of analyzed feed samples were 60.0%–100%. Taken together, these results remind us that, 1) the serious situation of mycotoxin contamination in feed requires strict supervision, 2) it is desperately necessary to constant monitoring and continue research effort on the prevention and mitigation of co-contamination of mycotoxin due to a considerable number of feed samples containing more than one mycotoxin, and 3) suitable and targeted strategies controlling mycotoxin occurrence and toxicity need to be researched and applied.

## Data Availability

The datasets used and/or analyzed during the current study are publicly available.

## Abbreviations

AFB_1_: Aflatoxin B_1_
DDGS: Dried distillers grains with solubles
DON: Deoxynivalenol
LOD: Limit of detection
ZEN: Zearalenone
